# 426. COVID-19 Infection Prevention Practices That Exceed CDC Guidance: Balancing Extra Caution Against Impediments to Care

**DOI:** 10.1093/ofid/ofab466.626

**Published:** 2021-12-04

**Authors:** Shruti K Gohil, Edward Septimus, Kenneth Sands, Eunice J Blanchard, Julia Moody, Annabelle De St Maurice, Deborah S Yokoe, Deborah S Yokoe, Jennie H Kwon, Jonathan Grein, Stuart H Cohen, Daniel Uslan, Vasudev Milind, Shannon C Mabalot, Micaela H Coady, Sljivo Selsebil, Kimberly N Smith, Brandon Carver, Russell Poland, Jonathan B Perlin, Richard Platt, Susan S Huang

**Affiliations:** 1 UC Irvine School of Medicine, Irvine, California; 2 Harvard Medical School, Houston, TX; 3 HCA Healthcare, Nashville, TN; 4 University of California, Los Angeles, Los Angeles, CA; 5 University of California, San Francisco, San Francisco, CA; 6 Washington University School of Medicine, St. Louis, Missouri; 7 Cedars-Sinai Medical Center, Los Angeles, CA; 8 University of California, Davis, Sacramento, CA; 9 UCLA, Los Angeles, CA; 10 University of California, Irvine School of Medicine, Irvine, CA; 11 Sharp Memorial Hospital, San Diego, CA; 12 Harvard Pilgrim Healthcare Institute, Boston, Massachusetts; 13 University of California, Irvine , Irvine, CA

## Abstract

**Background:**

At the outset of the COVID-19 pandemic, healthcare workers (HCWs) raised concerns about personal risks of acquiring infection during patient care. This led to more stringent infection prevention practices than CDC guidelines during a time of uncertainty about transmission and limited U.S. testing capacity. Hospitals were challenged to protect against true COVID-19 exposure risks, while avoiding use of unproven measures that could interfere with timely, high quality care. We evaluated hospital experiences with HCW COVID-19 exposure concerns impacting clinical workflow/management.

**Methods:**

We conducted a 32-question structured survey of hospital infection prevention leaders (one per hospital) from CDC Prevention Epicenters, University of California (CA) Health system, HCA Healthcare, and the Southern CA Metrics Committee between May–Dec, 2020. We assessed facility characteristics and COVID-19 exposure concerns causing changes in respiratory care, procedure delays/modifications, requests to change infection prevention processes, disruptions in routine medical care, and health impacts of PPE overuse. Percentages were calculated among respondents for each question.

**Results:**

Respondents represented 53 hospitals: 22 (42%) were small (< 200 beds), 14 (26%) medium (200-400 beds), and 17 (32%) large ( >400 beds) facilities. Of these, 11 (21%) provided Level 1 trauma care, and 22 (41%) provided highly immunocompromised patient care; 75% had cared for a substantial number of COVID-19 cases before survey completion. Majority reported changes in respiratory care delivery (71%-87%), procedural delays (75%-87%), requests to change infection prevention controls/protocols (58%-96%), and occupational health impacts of PPE overuse including skin irritation (98%) and carbon dioxide narcosis symptoms (55%) (Table).

**Conclusion:**

HCW concerns over work-related COVID-19 exposure contributed to practice changes, many of which are unsupported by CDC guidance and resulted in healthcare delivery delays and alterations in clinical care. Pandemic planning and response must include the ability to rapidly develop evidence to guide infection prevention practice.

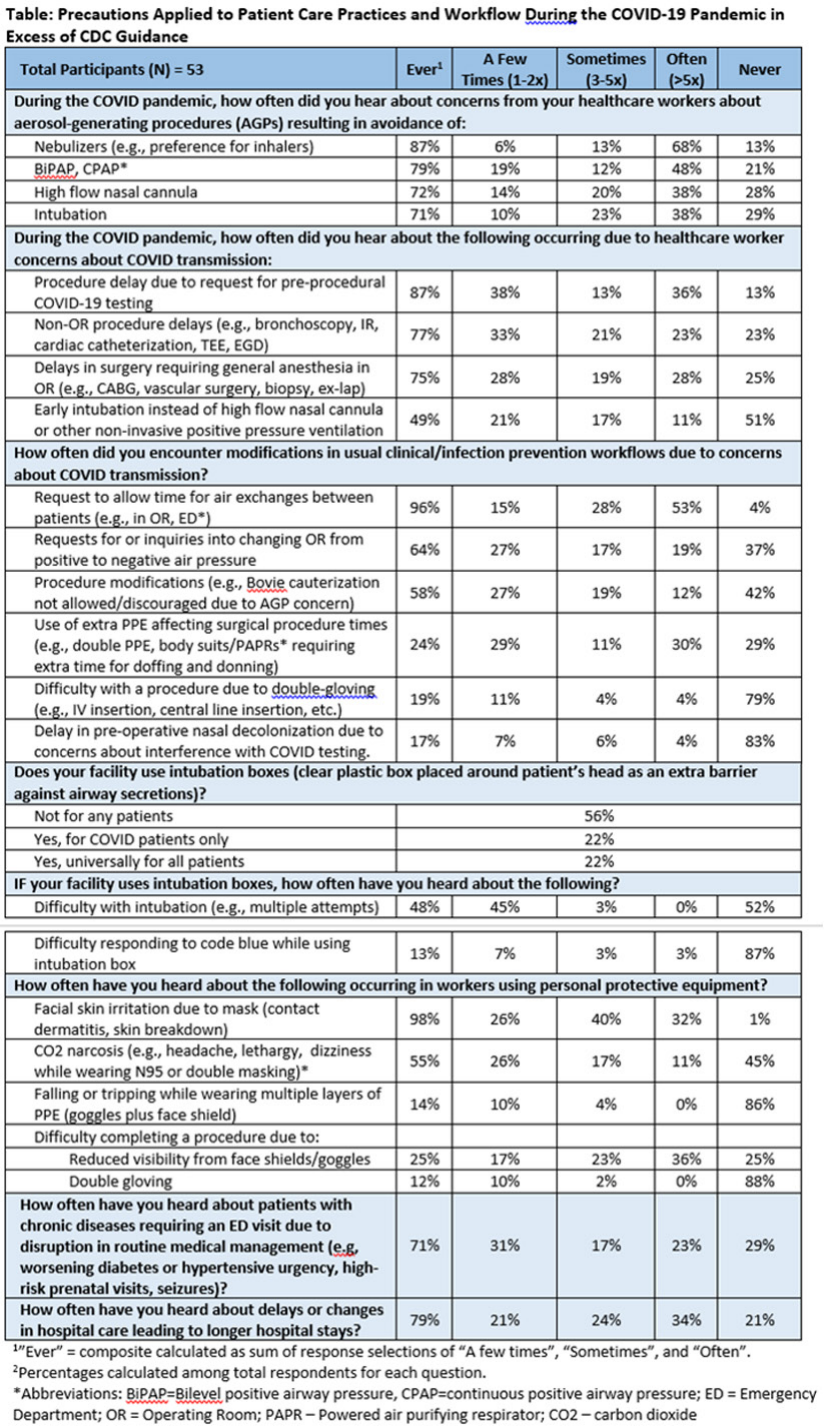

**Disclosures:**

**Shruti K. Gohil, MD, MPH**, **Medline** (Other Financial or Material Support, Co-Investigator in studies in which participating hospitals and nursing homes received contributed antiseptic and cleaning products)**Molnycke** (Other Financial or Material Support, Co-Investigator in studies in which participating hospitals and nursing homes received contributed antiseptic and cleaning products)**Stryker (Sage**) (Other Financial or Material Support, Co-Investigator in studies in which participating hospitals and nursing homes received contributed antiseptic and cleaning products) **Edward Septimus, MD**, **Medline** (Other Financial or Material Support, Conducted studies in which participating hospitals received contributed antiseptic products)**Molnlycke** (Other Financial or Material Support, Conducted studies in which participating hospitals received contributed antiseptic products) **Kenneth Sands, MD, MPH**, **Medline** (Other Financial or Material Support, Conducted studies in which participating hospitals received contributed antiseptic product) **Eunice J. Blanchard, MSN RN**, **Medline** (Other Financial or Material Support, Conducted studies in which participating hospitals received contributed antiseptic product) **Julia Moody, MS**, **Medline** (Other Financial or Material Support, Conducted studies in which participating hospitals received contributed antiseptic product)**Molnlycke** (Other Financial or Material Support, Conducted studies in which participating hospitals received contributed antiseptic product) **Deborah S. Yokoe, MD, MPH**, Nothing to disclose **Jonathan Grein, MD**, **Gilead** (Other Financial or Material Support, Speakers fees) **Stuart H. Cohen, MD**, **Seres** (Research Grant or Support) **Kimberly N. Smith, MBA**, **Medline** (Other Financial or Material Support, Conducted studies in which participating hospitals received contributed antiseptic product) **Brandon Carver, BA**, **Medline** (Other Financial or Material Support, Conducted studies in which participating hospitals received contributed antiseptic product) **Russell Poland, PhD**, **Medline** (Other Financial or Material Support, Conducted studies in which participating hospitals received contributed antiseptic product) **Jonathan B. Perlin, MD, PhD**, **Medline** (Other Financial or Material Support, Conducted studies in which participating hospitals received contributed antiseptic product)**Molnlycke** (Other Financial or Material Support, Conducted studies in which participating hospitals received contributed antiseptic product) **Richard Platt, MD, MSc**, **Medline** (Research Grant or Support, Other Financial or Material Support, Conducted studies in which participating hospitals received contributed antiseptic product)**Molnlycke** (Other Financial or Material Support, Conducted studies in which participating hospitals received contributed antiseptic product) **Susan S. Huang, MD, MPH**, **Medline** (Other Financial or Material Support, Conducted studies in which participating hospitals and nursing homes received contributed antiseptic and cleaning products)**Molnlycke** (Other Financial or Material Support, Conducted studies in which participating hospitals and nursing homes received contributed antiseptic and cleaning products)**Stryker (Sage**) (Other Financial or Material Support, Conducted studies in which participating hospitals and nursing homes received contributed antiseptic and cleaning products)**Xttrium** (Other Financial or Material Support, Conducted studies in which participating hospitals and nursing homes received contributed antiseptic and cleaning products)

